# Microbial community profiling of ammonia and nitrite oxidizing bacterial enrichments from brackishwater ecosystems for mitigating nitrogen species

**DOI:** 10.1038/s41598-020-62183-9

**Published:** 2020-03-23

**Authors:** Viswanathan Baskaran, Prasanna K. Patil, M. Leo Antony, Satheesha Avunje, Vinay T. Nagaraju, Sudeep D. Ghate, Suganya Nathamuni, N. Dineshkumar, Shankar V. Alavandi, Kizhakedath K. Vijayan

**Affiliations:** 1 Aquatic Animal Health and Environment division, ICAR-Central Institute of Brackishwater Aquaculture, Santhome High road, Chennai, 600028 India; 2 Yenepoya Research Centre, Yenepoya University, Mangalore, 575018 India

**Keywords:** Metagenomics, Metagenomics, Element cycles, Element cycles

## Abstract

Nitrogen species such as ammonia and nitrite are considered as major stressors in modern aquaculture practices. We developed enrichments of ammonia oxidising bacteria (AOB) and nitrite oxidising bacteria (NOB) for effective mitigation of nitrogenous wastes in the shrimp culture operations. The objective of this study was to understand the microbial community composition of AOB and NOB enrichments using the V3-V4 region of the 16S rDNA gene by Illumina MiSeq sequencing. The analysis revealed 2948 and 1069 OTUs at 97% similarity index and Shannon alpha diversity index of 7.64 and 4.85 for AOB and NOB enrichments, respectively. Comparative analysis showed that a total of 887 OTUs were common among AOB and NOB enrichments. The AOB and NOB enrichment were dominated by Eubacteria at 96% and 99.7% respectively. Proteobacterial phylum constituted 31.46% (AOB) and 39.75% (NOB) and dominated by α-*Proteobacteria* (20%) in AOB and γ-*Proteobacteria* (16%) in NOB. Among the species in AOB enrichment (2,948) two sequences were assigned to ammonia oxidising bacterial group belonging to *Nitrosomonas*, and *Nitrosococcus* genera and two belonged to archaeon group comprising *Nitrosopumilus* and Candidatus *Nitrososphaeraea* genera. The NOB enrichment was predominated by *Nitrospiraceae* and *Thermodesulfovibrionaceae*. Further, the data revealed the presence of heterotrophic bacteria contributing to the process of nitrification and form microcosm with the AOB and NOB. PICRUSt analysis predicted the presence of 24 different nitrogen cycling genes involved in nitrification, denitrification, ammonia and nitrogen transporter family, nitrate reduction and ammonia assimilation. The study confirms the presence of many lesser known nitrifying bacteria along with well characterised nitrifiers.

## Introduction

Aquaculture is an important economic activity supplying quality animal protein, generating employment and providing foreign exchange. Fish and fishery products are the most traded food items in the world, and an estimated 45% of the fish produced enters the international market. In terms of value, shrimp/prawn is the second most traded item next only to salmon in the USD 152 billion global seafood market^1^. In the modern-day intensive and semi-intensive shrimp aquaculture, management of accumulating metabolic wastes, especially in zero water exchange systems has been a major challenge. Accumulation of nitrogenous wastes generated by animal excreta and degradation of uneaten feed leads to deterioration of culture environment and stress to farmed animals^2–4^. Ammonia is the primary end product of protein metabolism in most aquatic animals^5^ and is also produced following microbial decomposition of organic wastes. Increase in the levels of nitrogenous species in the shrimp haemolymph leads to reduced food intake, increased oxygen consumption, increased excretion of nitrogen, and altered protein concentrations cause moderate to high mortality^6^. Further, the ammonia (>5 ppm) and nitrite (>0.35 ppm) are known to affect immunity and enhance susceptibility to diseases. Nitrite, an intermediary in the nitrogen metabolism, transforms the haemocyanin to meta-haemocyanin, reducing its ability to transfer oxygen to the tissues leading to hypoxia and death^7^.

Nitrification is the biological oxidation of ammonia (NH_3_) to nitrite (NO_2_^−^) and then to nitrate (NO_3_^−^), is a major oxidative process involved in maintenance of global nitrogen cycle in aquatic systems^8^ and soil^9^. This process is predominantly carried out by two different classes of lithoautotrophic microbes, namely the ammonia-oxidising bacteria (AOB) (e.g., *Nitrosomonas* and *Nitrosospira*) and the ammonia-oxidising archaea (AOA) (e.g., *Nitrososphaerea*, *Nitrosopumilus*) as one guild that convert ammonia to nitrite. Key enzymes involved in the process are ammonia monooxygenase encoded by the amoCAB genes and hydroxylamine dehydrogenase encoded by the haoAB genes^10^. The nitrite-oxidising bacteria (NOB) (e.g., *Nitrospira* and *Nitrobacter*) convert nitrite to nitrate mediated by the enzyme nitrite oxidoreductase encoded by nxrAB genes^11^. Recent studies have shown that nitrification is also carried out by heterotrophic bacteria which are commonly called as heterotrophic nitrifying bacteria^12–14^; however, they are lesser efficient than the autotrophs^15^.

In the intensive aquaculture system, sustainable management of toxic nitrogenous metabolites plays a crucial role in assured production. Generally, these metabolites are controlled by the application of adsorbents like Yucca extract and zeolite^16,17^ or by microbial interventions. The utility of adsorbents is restricted to physical removal of the ammonia build up in the system and needs repeated application. On the other hand, a recent study doubts the efficiency of mixotrophic microbes in effective mitigation of nitrogenous wastes in aquaculture^18^. However, factors like microbial diversity, salinity tolerance, conversion efficiency, growth rate and enrichment could influence the performance of formulated microbial consortia. Though AOB and NOB are efficient in nitrification, their slow-growing nature has been a hindrance in formulating a microbial product for bioremediation^19,20^. We have developed a microbial enrichment with nitrification property and the efficacy was tested in the laboratory and brackishwater aquaculture farms. The objective of this paper is to understand the microbial diversity of the enrichments developed in the laboratory using 16S rDNA sequencing analysis.

## Results and Discussion

**Figure 1:**
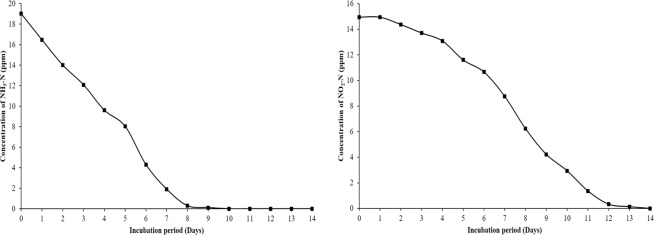
*In-vitro* Ammonia oxidation rate (**a**) and Nitrite oxidation rate (**b**) by AOB and NOB enrichments.

AOB and NOB cultures were enriched from the pond sediments under autotrophic conditions with ammonia and nitrite as a sole energy source. For determining *in vitro* activity of the consortia, the established enrichments were spiked with known concertation of ammonia (19 ppm) and nitrite (16 ppm) as described in methods. The AOB enrichment efficiently oxidised ammonia to nitrite in 10 days while NOB consortium oxidized nitrite to nitrate in 14 days (Fig. 1). Though non-specific isolation media is available, it is a challenging effort to isolate slow-growing nitrifying bacterial species in pure form and are maintained as enrichments. Our enrichments being highly efficient in the oxidation of ammonia and nitrite, in the present study, 16S rRNA high throughput sequence methods were used for knowing the bacterial diversity in the AOB and NOB consortia, and the results revealed the presence of several unclassified lineages in these enrichments.

### Diversity of microbial communities

**Figure 2:**
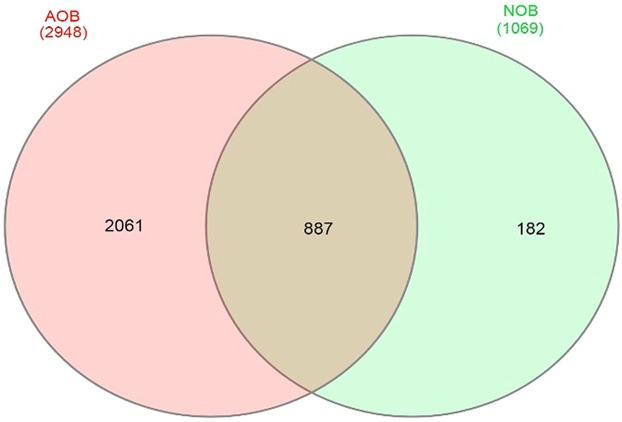
Venn diagram showing number of common OTUs between AOB and NOB consortia.

In total, approximately 488,402 bacterial sequence tags from AOB enrichments and 384,579 bacterial sequence tags from NOB enrichments for 16S rDNA with an average length of 2 × 300 bp were obtained. Following sub-sampling, a total of 2,948 OTUs for AOB and 1,069 OTUs for NOB consortia respectively were obtained at 97% similarity index. Shannon alpha diversity index was found to be 7.64 in AOB enriched culture and 4.85 in NOB consortia confirming rich microbial diversity among the enrichments (Table 1). Comparative analysis showed that a total of 887 (21.92%) OTUs were common among AOB and NOB consortia (Figs. 2, S1).

**Table 1:** Alpha diversity Index of microbial consortia involved in ammonia and nitrite oxidation.

Diversity index	AOB consortium	NOB consortium
Shannon	7.64	4.85
ACE	1064.9	1503.3
Chao1	1075.8	1492.2
Simpson	0.95	0.94
Fisher	149.5	155.7

### Relative abundance of bacterial community

**Figure 3:**
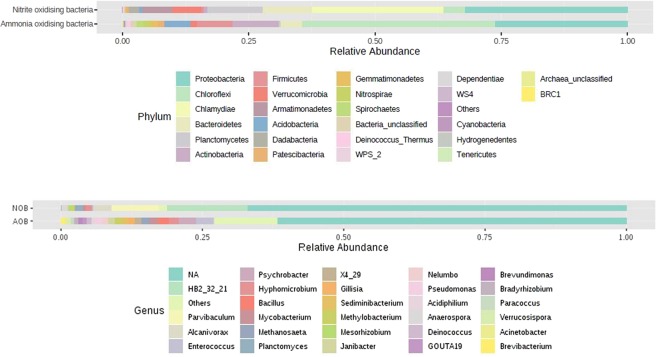
Phylum (**a**) and genus (**b**) level distribution among AOB and NOB enrichments.

**Figure 4:**
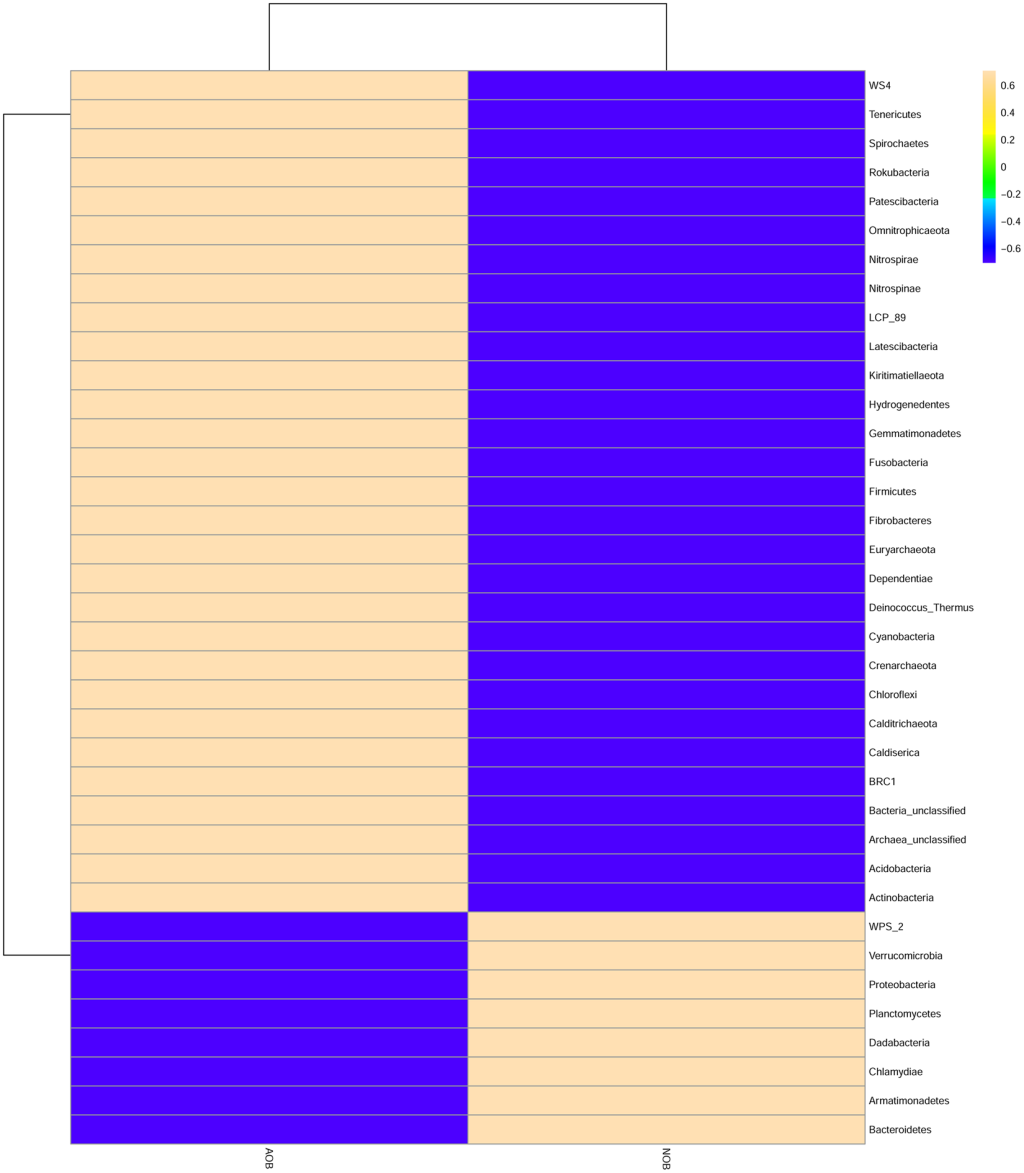
Dominant taxa present in AOB and NOB consortia at the phylum level.

With 16S rDNA high throughput sequencing, nearly 4,000 OTUs were recovered from the two-distinct nitrifying bacterial enrichments. Altogether a total of 47 bacterial phyla were observed in both the enrichments. Eubacteria dominated both AOB and NOB consortia (96% and 99% respectively) with remaining taxa belonging to Archaea. The proteobacterial phylum was found to be dominant in both the enrichments, with 31.46% and 39.75% in AOB and NOB consortia respectively. Additionally, *Acidobacteria, Actinobacteria, Bacteroidetes, Chloroflexi, Cyanobacteria, Firmicutes, Nitrospira* and *Planctomycetes*were were the other phyla present in both AOB and NOB enrichments. Among the proteobacterial phylum, α-Proteobacteria dominated in AOB (20%) and NOB (18%) consortia while γ proteobacteria constituted 16% and 8% in NOB and AOB consortia respectively (Figs. 3a, 4).

### Taxonomic composition of microbial enrichments

#### Microbial communities in ammonia oxidising enrichment

**Figure 5:**
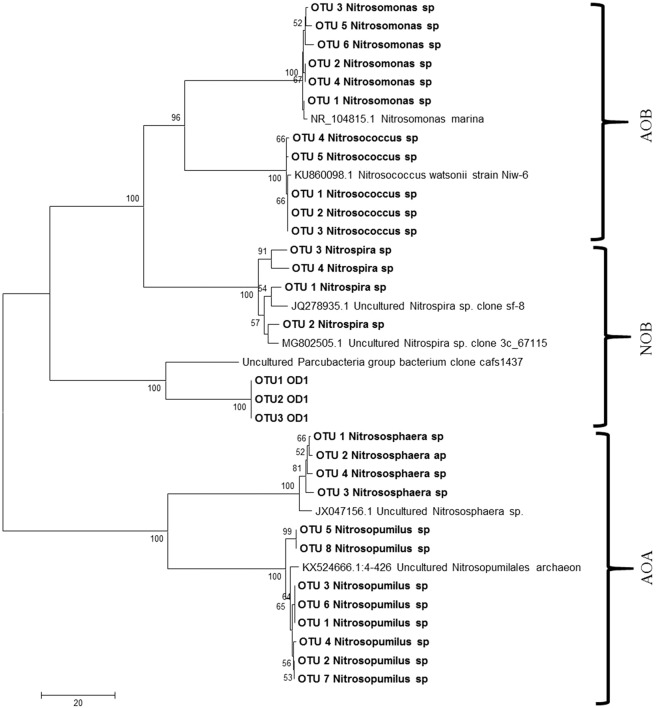
Phylogenetic analysis of representative sequences of Ammonia oxidising bacteria, archaea and nitrite oxidising bacteria in AOB and NOB enrichments.

A total of 2,948 species were observed in AOB consortia, out of which two sequences were assigned to AOB group belonging to *Nitrosomonas*, and *Nitrosococcus* genera and two belong to archaeon group comprising *Nitrosopumilus* and *Nitrososphaera* genera. Of the OTUs belonging to AOB guild, 20 OTUs were identified as *Nitrosomonas marina*. The enrichments also contained 107 OTU s of *Nitrosococcus* genera (Fig. 5), represented under unclassified species clade. Among the ammonia oxidising archaea group, eight OTUs belong to *Nitrosopumilus* comprising unclassified species and four OTUs belonging to *Nitrososphaeraea* comprising the Candidatus *Nitrososphaeraea gargensis* (Fig. 5). The genus *Stanieria* belonging to the group of methyl ammonia oxidiser community were also found (four OTUs). Similarly, 269 OTUs showing similarity to *Nitrospira*-like AOB organisms were observed in the consortia. Genera with lesser abundance belonging to *Psychrobacter, Bacillus* (two OTUs), *Hyphomicrobium* (two OTUs), *Methanosaeta* (two OTUs), *Sediminibacterium* (one OTU), *Pseudomonas* (four OTUs) and Candidatus species (six OTUs) were recorded (Fig. 3b, Table S1).

In our study, two types of aerobic autotrophic microorganisms were observed in the AOB enrichments such as *Nitrospira* and *Nitrosomonas* that are known to be major players of ammonia oxidation. Autotrophic microbes can grow till exhaustion of nutrients and remain dormant for a certain period of time. Though heterotrophic microbes can grow five times faster than autotrophs, the latter group of microbes have significantly higher nitrification efficiency^21^. The AOB strains obtained in our study were assigned to the *Nitrosomonas* lineage (Fig. 5), confirming with the previous studies that these bacteria in this lineage can survive in low ammonia concentration, in soils^9^, lake sediments^22^ and biofilters^23^. Apart from ammonia oxidising bacterial population, archaea bacterial population were also detected in the developed enrichments. The majority of AOA classified till now were found to be oligotrophic and grow under low ammonia concentration. Gao *et al*.^24^ showed that AOB were more competitive than AOA under high concentration of ammonia. Similarly, substrate inhibition of archaeal nitrification under high concentration of ammonia has been reported^25–28^. Distribution of AOA is also ecosystem dependent, for example, Ca. *Nitrosopumilus* is widely present in seawater^29^ while Ca. *Nitrososphaera* more abundant in soil habitats with a wide range of pH and also in different aquatic ecosystems^30^. The results obtained in this study also showed lower diversity of AOA than AOB confirming with the previous studies. Phylogenetic analysis of archaea bacterial sequences were clustered into *Nitrosopumilus* and *Nitrosophaera* group and showed a distinct lineage towards ammonia oxidising bacterial groups (Fig. 5).

From our findings, the majority of microbes identified were from Proteobacteria phylum that is widely present in natural environments and plays a vital role in nutrient cycling and mineralisation of organic compounds^31^. Nearly 62% of the identified sequences were found to be unclassified genera (Fig. S2) indicating there might be novel microbial genera involved in ammonia oxidation or involved in aiding ammonia oxidation process. The dominant genera of ammonia-oxidising nitrifiers such as *Nitrosomonas* sp., *Nitrosomonaseuropea* and *Nitrospira* sp. have been documented in this study are responsible for efficient nitrification. Members of the group *Nitrospira* which is known to be a complete ammonia oxidizer^32^ is also found in our enrichments.

The *Limnohabitans* spp. present in AOB consortia is well-studied opportunistic bacterial group, with rapid generation time, acting on low molecular weight dissolved organic matter^33^. A recent study showed *Limnohabitans* species, strains Rim28 and Rim47 had great metabolic versatility, including photosynthesis, autotrophic carbon fixation, and ammonium and sulphur oxidisation^34^. Fitzgerald *et al*.^35^ reported the involvement of *Luteibacter* spp. of Xanthomonadaceae family in autotrophic utilisation of ammonia as a sole source of nitrogen in the low DO-nitrification process. Along with *Nitrosomonas*, *Nitrosopumilus* and *Limnohabitans* in AOB consortia these groups can also play a role in oxidising ammonia into nitrite.

Heterotrophic bacterial cultures like *Bacillus*, *Arthrobacter*, *Pseudomonas* and *Exiguobacterium* belonging to various phyla such as Firmicutes, Actinobacteria and Proteobacteria were observed in the enrichments and may not specifically be ammonia-oxidising nitrifiers; probably from the source water used for raising the enrichment. The results obtained in our study show that the enrichments consist of bacteria having mixotrophic ammonia oxidation and are well corroborated with the previous studies^36^ (Fig. S3).

### Microbial communities in nitrite-oxidising enrichments

A total of 1,069 OTUs were observed in the NOB enrichments of which 184 OTUs belonging to phylum Nitrospirae were detected predominantly, two families belonging to Nitrospiraceae and Thermodesulfovibrionaceae. Under Nitrospiraceae 56 OTUs belong to unclassified *Nitrospira* genera whereas, 36 OTUs of Thermodesulfovibrionaceae families were distributed into three unclassified genera. NOB enrichments had 60% of unclassified sequences (Fig. S2). One of the interesting findings in NOB consortia is the presence of superphylum OD1. A total of 17,961 sequences belonging to the phylum OD1 has been classified as superphylum Candidate Phyla Radiation (CPR) Parcubacteria (Fig. 5) group which was identified as a major player in marine nitrogen cycle^37^.

In this study, we have documented a diverse lineage of Nitrospirae phylum known to play a major role in the nitrite oxidising process. To the best of our knowledge, a few chemolithoautotrophic microbes with nitrite oxidation have been documented. Most of these groups were previously documented to play a major role in nitrite oxidation and also involved in the denitrification process with key genes involved (Table S1). For the nitrite oxidisers, eight OTUs related to *Nitrospira* were detected in the enrichment. *Nitrospira* (Fig. 4) is identified as the most abundant nitrite oxidisers in low-nitrite environments^38,39^. A poorly characterised taxon, 0319-6A21 (Table S1) belongs to *Nitrospira* was first reported from lava caves than in surface soils^40^. Organic compounds produced by both AOB and NOB serve as a substrate for heterotrophic microbial growth^41^ whereas, a *Nitrospira* population can feed on the dead heterotrophic community during the enrichment in mineral nitrite medium^42^. NOB may also benefit from the presence of heterotrophs, for example, via heterotrophic nitrate reduction.

The previous study has demonstrated that members of Proteobacteria can utilise reduced sulphur compounds to obtain energy and also through carbon assimilation via the reductive tricarboxylic acid (rTCA) cycle^43^. Interestingly rare taxa <1% belong to Armatimonadetes, AC1, NC10, NKB19, OP8 and GN04 (Table S1) were detected in both the enrichments. Presence of NC10 group in both the samples suggests their involvement in methane oxidation coupled with nitrification process^44,45^ (Fig. S3). Although these groups were found to be present in less number, previous reports have also predicted their involvement in nitrogen cycle coupled to some other biogeochemical pathways.

### Heterotrophic nitrate-reducing bacterial communities

The traditional theory of biological nitrogen removal makes a rigid difference between nitrification and denitrification process based on distinct growth conditions of nitrifiers and denitrifiers; however, heterotrophic nitrification and aerobic denitrification processes can simultaneously occur^46,47^. The potential for nitrification and denitrification was detected in both autotroph and heterotroph microbial lineages, suggesting the involvement of a diverse range of nitrogen metabolic pathway. Heterotrophic nitrifying bacteria produce hydroxylamine, nitrite and nitrate by nitrification process using organic carbon as a source for their growth. Most of these bacteria are capable of converting nitrification products directly to nitrogen gas through the process of aerobic denitrification^48^. In this study other than autotrophic microbes, heterotrophic microbes were also predominantly present in both the enrichments. Proteobacteria were the most critical contributors of all genes involved in denitrification pathway, besides the presence of other groups like Nitrospirae, Bacteroidetes, and uncultured microorganisms were shown to play essential roles in denitrification. The dominant denitrifiers in our enrichments mainly include *Alcaligenes*, *Pseudoxanthomonas, Pseudomonas, Marinobacter, Shewanella, Thalassospira* and *Rhodobacter* (Table. S1), most of which are taxonomically affiliated with ß or γ proteobacteria and bacteroidetes^49^. Proteobacterial groups, found in both the enrichments, were closely related to *Hyphomicrobium*, *Paracoccus*, *Pseudomonas* and *Comamonas* spp. (Table S1, Fig. S3) and known to be denitrifiers^50^. Studies by Feng *et al*.^51^ showed that bacteria belong to *Bacillus, P. putida, P. stutzeri, Hydrogenophaga*, and *Achromobacter* have been shown to have nitrification and aerobic denitrification abilities

**Figure 6:**
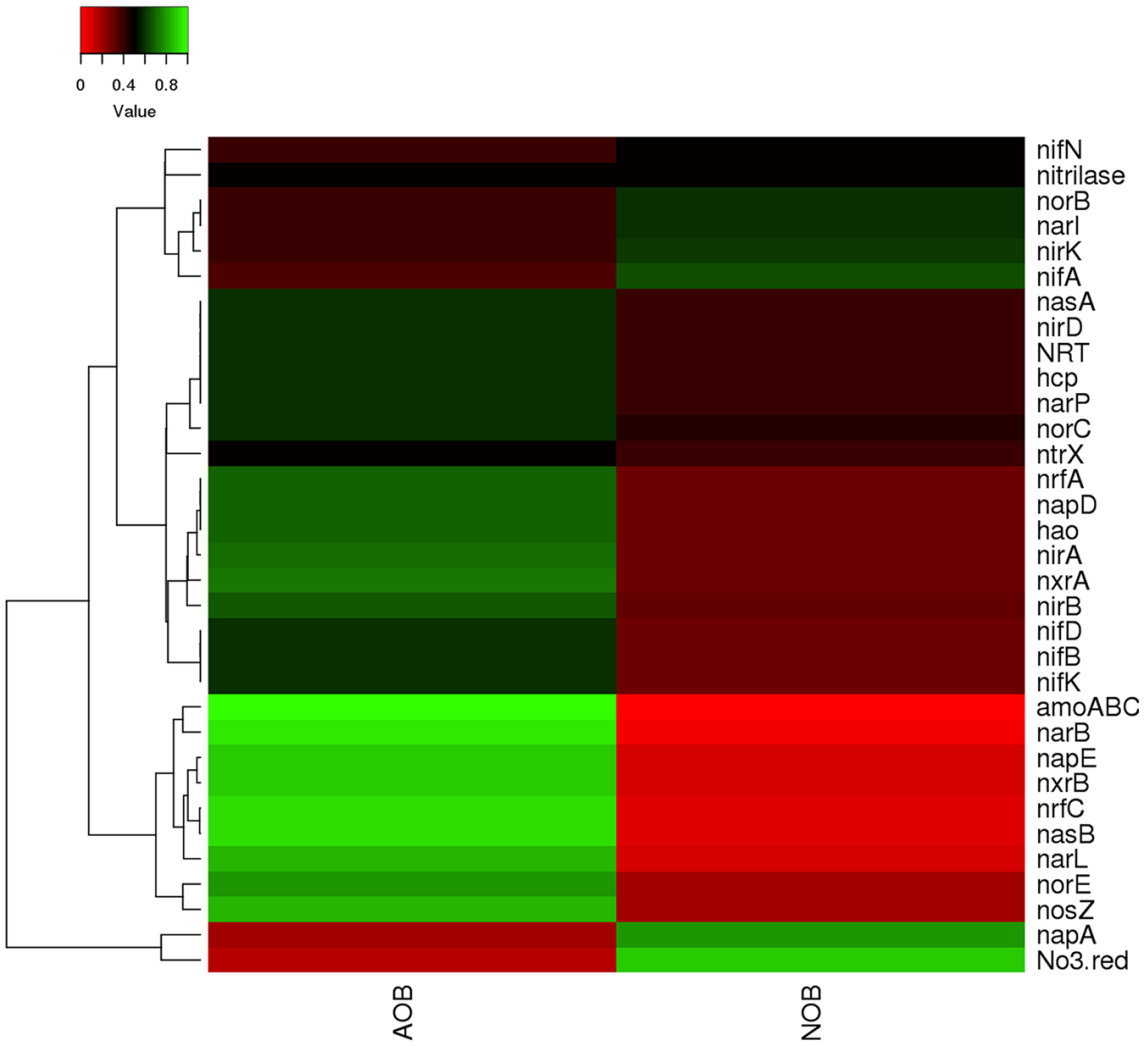
Heat map showing the distribution of genes involved in nitrogen cycle based on KEGG orthologous gene identification by PICRUSt analysis.

The heterotrophic genera identified in AOB enrichment belong to *Burkholderia*, *Exiguobacterium*, *Methanoregula* and *Methanosaeta* while, groups belonging to *Aequorivita*, Candidatus *Entotheonella*, *Erythrobacter*, *Flavobacterium*, *Idiomarina*, *Mesorhizobium*, *Oleibacter*, *Parvibaculum* were present in NOB enrichments. Groups belonging to *Alcanivorax, Anaerospora, Arenibacter, Bacillus, Bradyrhizobium, Brevibacterium, Brevundimonas*, Candidatus *Solibacter, Desulfovibrio, Devosia*, *Halorhodospira, Hyphomicrobium, Lewinella, Planctomyces, and Sphingobium* were present in both AOB and NOB enrichments (Table S1, Fig. S3). These groups present in enrichments were classified based on their predicted functional properties such as denitrification, nitrification and nitrate reduction using KEGG orthology database (Fig. 6, Table S1). In this study, a broader diversity of metabolic genes involved in nitrogen metabolism, *viz*. nitrification, denitrification, nitrogen fixation, and dissimilatory nitrate reduction to ammonia (DNRA) were predicted using PICRUSt analysis in both the AOB and NOB enrichments.

Many OTU’s belong to yet to be classified group described as Candidatus species were also found to be predominant in both AOB and NOB consortia. Based on recent findings, AOB and NOB enrichments contain bacterial isolates belonging to super-phylum Verrucomicrobia, which are involved in methane regulated pathway^52^ were documented in both the enrichments. Some of the notable groups, such as *Natronococcus*, known for nitrate reduction were present in both the enrichments. Other Candidatus groups belong to unclassified *Lautropia*, phylum BRC2, *Koribacter*, *Solibacter, Aquiluna*, and *Entotheonella* (Table S1)^12,53^ were also found to be denitrifier strains. Few groups belong to *Methanoregula*, and Candidatus *Hydrogenedens*^54^ were found to convert nitrate to ammonia through dissimilatory nitrate reduction pathway.

16S rDNA high throughput gene sequencing indicated that the nitrifying populations in the enrichment involve a *Nitrosomonas*-like *Nitrospira-*like AOB, *Nitrosopumilus*, Candidatus *Nitrososphaera* and *Nitrospira*-like NOB that are adapted to ammonia and nitrite condition, respectively. Previous studies have also reported the heterotrophic nitrification and ammonification pathway present in these groups, but their mode of function remains less known. Based on preferential enrichment, and the reports from existing literature, organisms belong to *Pseudomonas*, members of the family *Xanthomonadaceae*, *Limnohabitans*, and *Sphingomonas, CPR OD1*, Candidatus species of *Opitutus, Staneria*, and *Exiguobacterium* group have the potential to participate in ammonia as well as nitrite oxidation, can function either as heterotrophic nitrifiers, or via autotrophic nitrification through yet uncharacterized pathways^33,35,37^. The potential for nitrification and denitrification was detected in a diverse lineage of both autotroph and heterotroph microbial communities, suggesting a diverse range of potential involved in nitrogen metabolism. One such group observed in the enrichment is *Sulfurimonas*, which reduces nitrate to dinitrogen gas coupling sulphur oxidation to denitrification pathway has been previously documented by Cerqueira *et al*.^55^.

### Presumptive functional profile of microbes in the enrichments using PICRUSt analysis

**Figure 7:**
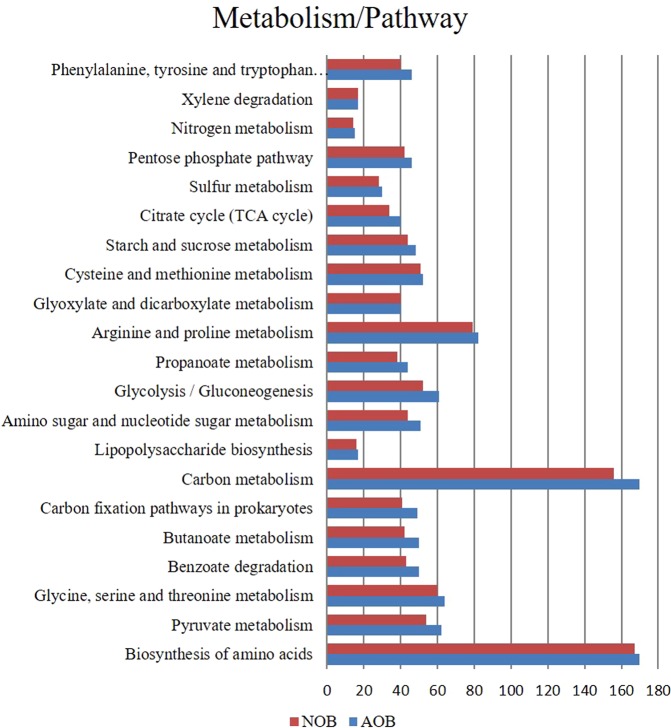
The general metabolic pathways of AOB and NOB consortiaby network enrichment analysis.

PICRUSt was used to predict the function of enrichments based on 16S rDNA and the functionalities obtained are predictive. The analysis predicted 24 different nitrogen cycling genes that are involved in nitrification, denitrification, ammonia and nitrogen transporter family, nitrate reduction and ammonia assimilation (Fig. 6, Table S1). Genes coding for the enzyme involved in nitronate monooxygenase, nitrile hydratase, nitrate reductase, nitrilase, nitric oxide dioxygenase, nitric oxide reductase, nitric-oxide synthase, nitrite reductase, nitric-oxide reductase, nitrogenase, nitric nitrogen fixation protein, nitroreductase/dihydropteridine reductase, nitrous-oxide reductase, nitroreductase, nitrate reductase, nitrogenase, nitric oxide reductase, which are distributed among several phyla were predicted. Most dominant gene cluster involved in nitrification, denitrification, DNRA and assimilatory nitrogen reduction is given in Table (S1). Other than nitrogen metabolism, metabolic pathways related to sulphur, carbon, propionate, TCA cycle, amino acid and sugar metabolism, biodegradation and carbon fixation pathways and pathways associated with the degradation of aromatic compounds, nitrotoluene degradation and polyaromatic hydrocarbon degrading groups were also predicted to be present in both the enrichments (Figs. 7 and S4). However, it is to be noted that use of function prediction tools relies mainly on the availability of reference genomes included in the algorithm^56^ and the predicted functions are indicative, which needs further confirmation.

Due to the presence of many unclassified genera in the enrichment, this study suggests that heterotrophic bacterial group documented in the enrichment may play a role in nitrogen conversion. From the findings, we believe presence of heterotrophic bacteria along with autotrophic bacterial groups and form a microcosm^57^. This may be due to production of the secondary metabolites, including nitrogenous compounds by autotrophs facilitating survival of the heterotrophic bacteria in the enrichments. Recently nitrification activity has been observed in heterotrophic bacteria, which are capable of utilising ammonia and nitrite^33,36,47^. Although nitrifying bacteria may act as primary producers and play a significant role in the nitrogen cycle, little is known about its adaptability with heterotrophic microbial community and mode of interactions.

## Conclusion

The AOB and NOB enrichments developed in our laboratory had efficient nitrification potential. The study has documented the microbes involved in the process of nitrification in the AOB and NOB enrichments. In addition to well-known autotrophs like *Nitrosomonas*, *Nitrosococcus*, *Nitrospira*, *Nitrosopumilus*, *Nitrososphaera;* many Candidatus species, heterotrophic bacteria such as *Marinobacter, Paracoccus, Burkholderia, Exiguobacterium, Alcaligenes, Pesudomonas*, *Hyphomicrobium*, *Caldilinea*, Thalassospira and *Rhodobacter* etc were observed in the enrichments. The present study also documented the presence of diverse lineage of phylum OD1 showing the importance of this group in the nitrogen cycle. The enrichments, beside containing ammonia and nitrite oxidising autotrophs were found to be associated with heterotrophic microbial groups involved in nitrification, denitrification and establishing a microcosm with DNRA and assimilatory nitrate reduction.

## Materials and method

### Microbial enrichments

Sediment samples (n = 66) were collected from shrimp aquaculture ponds (n = 15), having salinity 18–48 ppt from coastal regions of India. About 5 g from each sediment samples were aseptically inoculated into sterile Koops^58^ medium for AOB cultivation and Watson and Waterbury^59^ medium for NOB cultivation with pH 7.5–8.0 and incubated at 30 °C in an orbital shaker at 120 rpm for 24 days, and the culture has been continuously enriched for six months. Among the samples processed two enrichments were found to show promising activity and the efficient AOB and NOB enrichments were pooled separately. To understand the AOB and NOB oxidation rate, enrichments were spiked with a known concentration of ammonia (19 ppm) and nitrite (16 ppm). Daily sampling was conducted to analyse ammonia (by Nessler’s reagent^60^ at 405 nm) and nitrite (by Sulphanilamide, N-ethylene- di hydrochloride^61^ at 540 nm) oxidation rate.

### Preparation of MiSeq library

DNA was extracted from the AOB and NOB consortia by phenol: chloroform extraction protocol from Ghosh *et al*.^62^. The amplicon libraries were prepared using Nextera XT index kit (Illumina Inc. USA) as per metagenomic sequencing library preparation protocol. Primers for amplification of the 16S rDNA gene-specific for bacterial V3–V4 were designed at Eurofins Genomics and Bioinformatics laboratory (Bangalore, India) with the sequence as 341F 3′-GCCTACGGGNGGCWGCAG-5′ and 806R 3′-ACTACHVGGGTATCTAATCC-5′. Samples from two batches of culture for each of AOB and NOB were analysed for confirmation of the data.

### Data analysis

The raw sequencing data were processed using the QIIME pipeline v1.7.0 to obtain the high-quality tags and chimera checked using UCHIME algorithm against the reference database (Gold database) to generate clean data. Then the effective tags from all samples were clustered into operational taxonomic units (OTUs) using QIIME with 97% similarity. Taxonomy was assigned to the representative sequences using the RDP Classifier. The following diversity analyses were determined in QIIME (v1.7.0). The phylogenetic tree was constructed for representative sequences of predominant AOB and NOB enrichments using MEGA 7.

The sequences were also processed using a MOTHUR pipeline (v. 1.41.1) to filter reads for quality, create contigs and reduce noise^63^. Ambiguous sequences were discarded, and sequences longer than 466 base pairs (bp) were dislodged while those with a maximum homopolymer length of 6 bp were allowed. Sequences were aligned with SILVA database release 132, and the SILVA taxonomy was used for classification of representative sequences and operational taxonomic units (OTUs) at 97% similarity. Chimeras were identified and removed with the chimera.vsearch option. Alpha diversity (e.g., Chao, Shannon, Simpson, Fisher and ACE) for individual samples were estimated using MOTHUR and submitted to MicrobiomeAnalyst^64^. Samples analysed with MicrobiomeAnalyst were filtered for low abundance based on the mean abundance of OTUs, and for low variability using the inter-quantile range assessment.

### Functional pridiction

Metagenomic inference and functional analysis were performed using a phylogenetic investigation of communities by reconstruction of unobserved states (PICRUSt). Pathway analysis was conducted using the Kyoto Encyclopaedia of Genes and Genomes (KEGG) orthologous pathways. The 16S rDNA data were analysed as indicated by the PICRUSt genome prediction software (http://picrust.github.io/picrust/)^65^ from raw sequence reads in the following environment: NumPy (1.7.1), biom-format (1.3.1), PICRUSt (1.0.0-dev), and PICRUSt script (1.0.0-dev).

Functional predictions were assigned up to KO tier for metabolic pathways related to the nitrogen cycle, which are assumed to be more relevant for this study. Heat map clustering for genes involved in nitrogen cycle was done using heatmapper.ca^66^. Functional inference of microbiome was analysed from sequencing data through MOTHUR using the SILVA database, through Piphillin, a function prediction tool^56^. It uses the nearest-neighbour matching using global alignment between the 16S rRNA gene sequencing input OTUs representative sequences and abundance table with updated genomic databases to infer the metagenomics content of the samples. We used the web version of Piphillin incorporating the KEGG October 2018 version as a reference database, and applying 97% identity cut-off. The software uses USEARCH to generate genome abundance table which is then normalised by the copy number to get the genome content and is summed to get the KO relative abundance table. This output was analysed using MicrobiomeAnalyst tool for metabolic network visualisation and pathway mapping. The relative abundance of functional categories as Kyoto Encyclopaedia of Genes and Genomes (KEGG)^67^ pathways were generated from OTU table of assigned taxa and were integrated and interpreted in KEGG pathway for nitrogen metabolism and visualized using VANTED (v.2.2.1)^68^.

## Supplementary information

Supplementary information.

## Data Availability

The raw sequencing data of AOB (SRX5815865) and NOB (SRX5815867) consortia are available under the project ID PRJNA542210 in NCBI database.
