# Supplementary material for: Microbial community profiling of ammonia and nitrite oxidizing bacterial enrichments from brackishwater ecosystems for mitigating nitrogen species
Source: Sci Rep. 2020 Mar 23;10:5201. doi: 10.1038/s41598-020-62183-9 (PMC7090006; doi:10.1038/s41598-020-62183-9)
Supplement: Supplementary file 1 — Supplementary information. [file 41598_2020_62183_MOESM1_ESM.pdf]

Microbial community profiling of ammonia and nitrite oxidizing bacterial enrichments from brackishwater ecosystems for mitigating nitrogen species

Viswanathan Baskaran[1], Prasanna Kumar Patil[1*], M. Leo Antony[1], Satheesha Avunje[1], Vinay T. Nagaraju[1], Sudeep D. Ghate[2], Suganya Nathamuni[1], Dineshkumar N[1], Shankar V. Alavandi[1], Vijayan K. Kizhakedath[1]

[1]Aquatic Animal Health and Environment division, ICAR-Central Institute of Brackishwater Aquaculture, Santhome
[2]Yenepoya Research Centre, Yenepoya University, Mangalore-575018

Corresponding Author: P. K. Patil,

Phone: +919941468356; Email: pkpatilvet@gmail.com; pkpatil@ciba.res.in

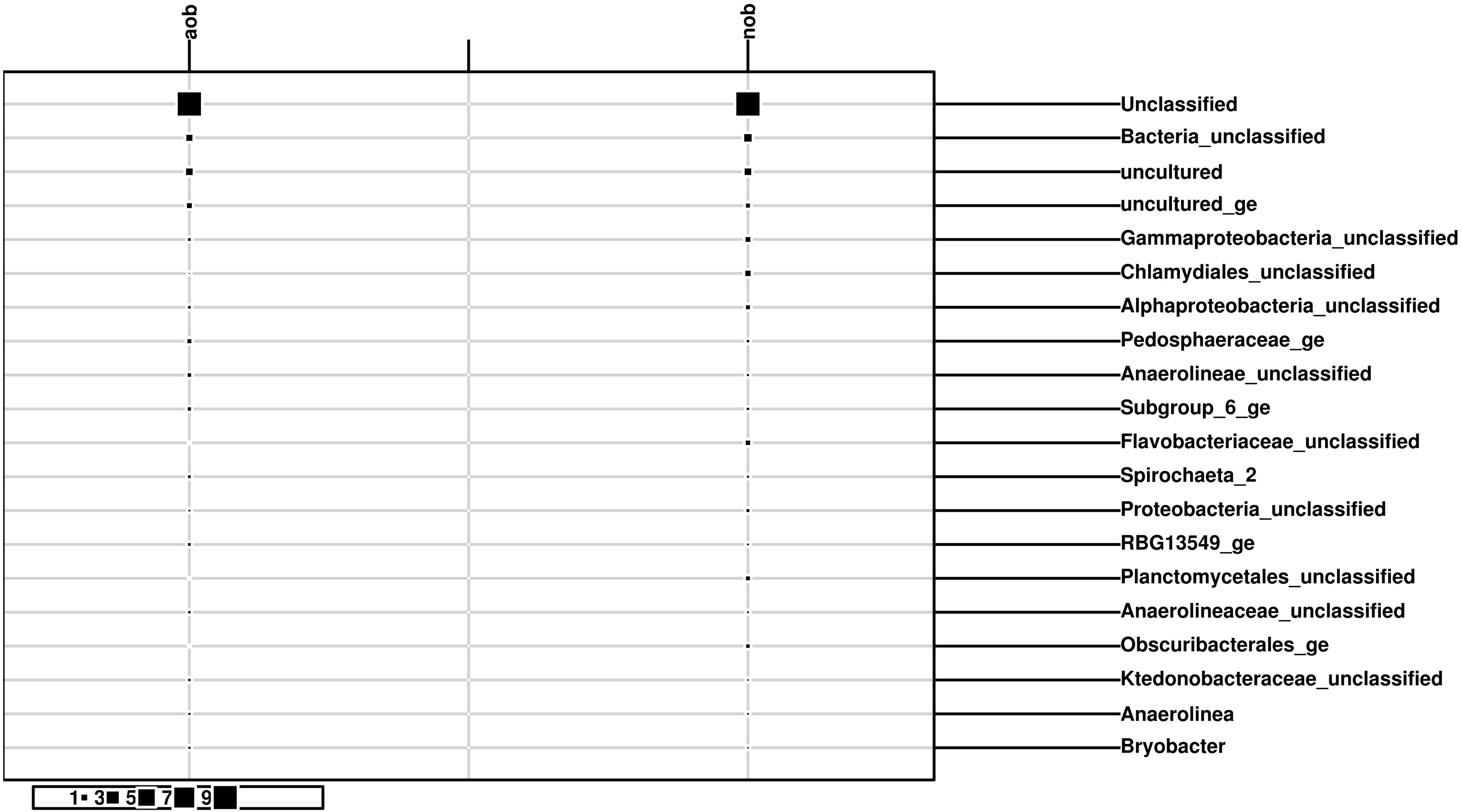

aob
nob
Unclassified
Bacteria_unclassified
uncultured
uncultured_ge
Gammaproteobacteria_unclassified
Chlamydiales_unclassified
Alphaproteobacteria_unclassified
Pedosphaeraceae_ge
Anaerolineae_unclassified
Subgroup_6_ge
Flavobacteriaceae_unclassified
Spirochaeta_2
Proteobacteria_unclassified
RBG13549_ge
Planctomycetales_unclassified
Anaerolineaceae_unclassified
Obscuribacterales_ge
Ktedonobacteraceae_unclassified
Anaerolinea
Bryobacter
1 3 5 7 9

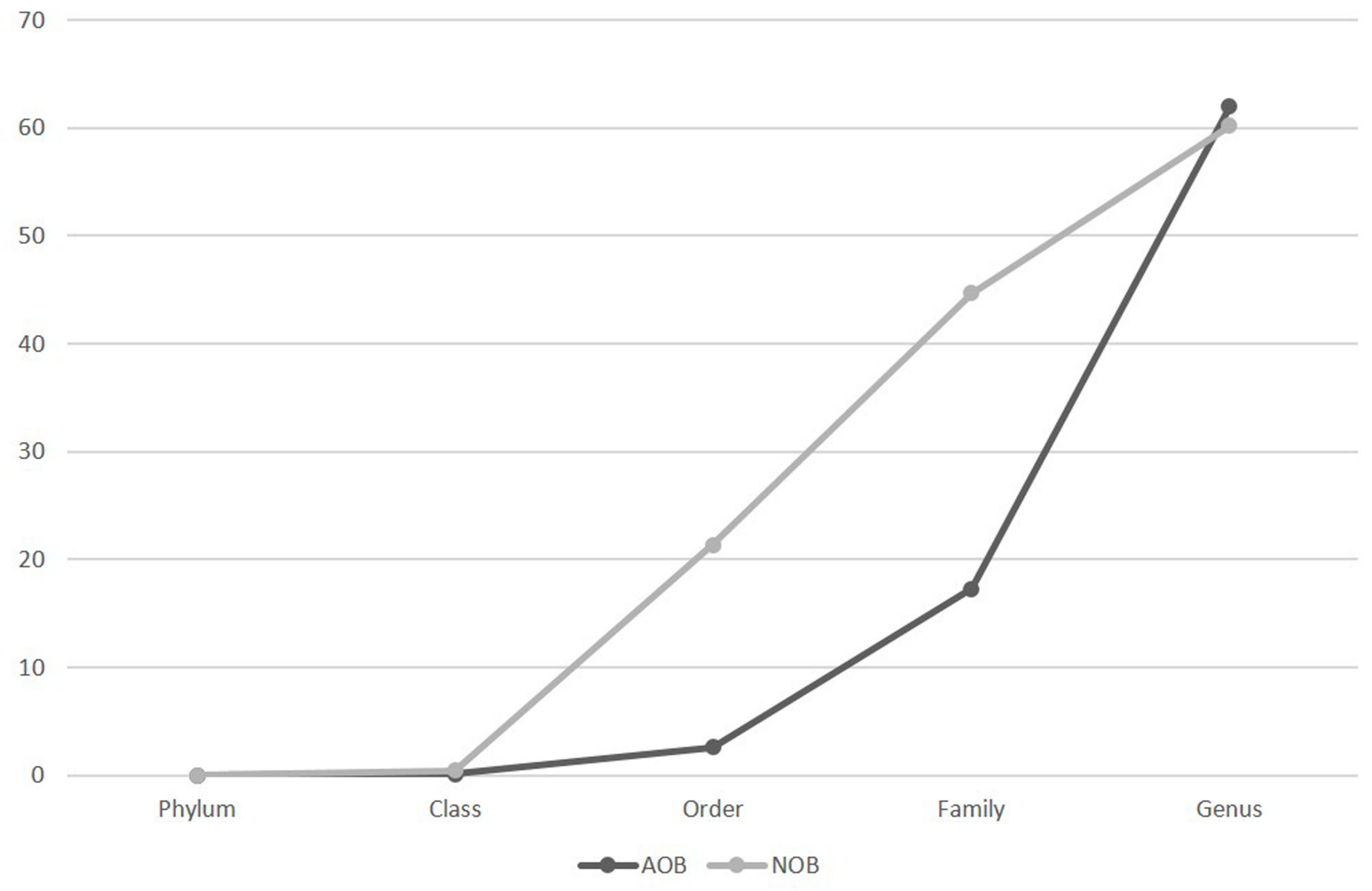
70
60
50
40
30
20
10
0
Phylum
Class
Order
Family
Genus
AOB
NOB

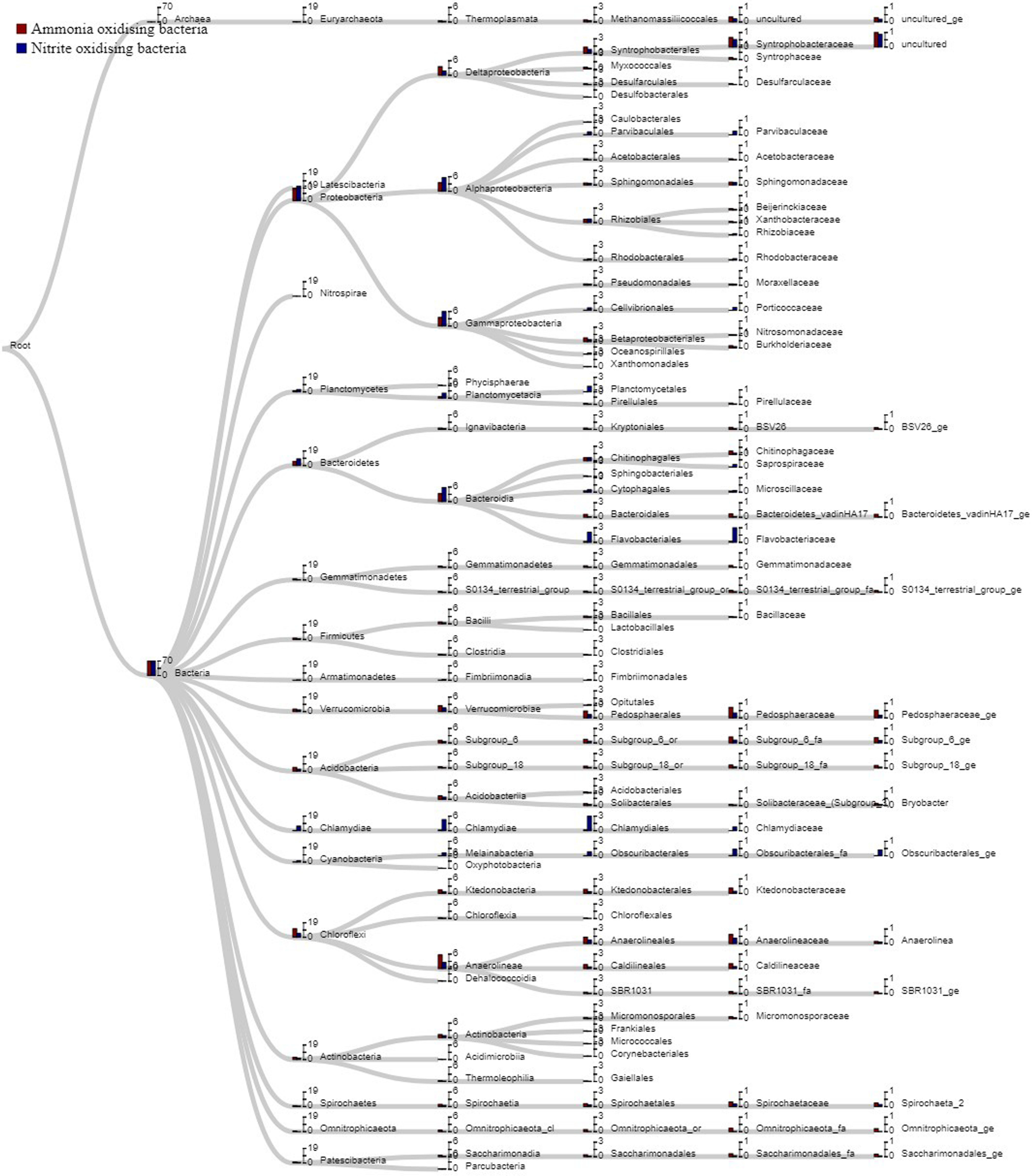

Ammonia oxidising bacteria
Nitrite oxidising bacteria
Root
Archaea
Euryarchaeota
Thermoplasmata
Methanomassiliicoccales
uncultured
uncultured_ge
Bacteria
Latescibacteria
Proteobacteria
Deltaproteobacteria
Syntrophobacterales
Syntrophobacteraceae
uncultured
Syntrophaceae
Myxococcales
Desulfarculales
Desulfarculaceae
Desulfobacterales
Alphaproteobacteria
Caulobacterales
Parvibaculales
Parvibaculaceae
Acetobacterales
Acetobacteraceae
Sphingomonadales
Sphingomonadaceae
Rhizobiales
Beijerinckiaceae
Xanthobacteraceae
Rhizobiaceae
Rhodobacterales
Rhodobacteraceae
Gammaproteobacteria
Pseudomonadales
Moraxellaceae
Cellvibrionales
Porticoccaceae
Betaproteobacteriales
Nitrosomonadaceae
Burkholderiaceae
Oceanospirillales
Xanthomonadales
Nitrospirae
Planctomycetes
Phycisphaerae
Planctomycetacia
Planctomycetales
Pirellulales
Pirellulaceae
Bacteroidetes
Ignavibacteria
Kryptoniales
BSV26
BSV26_ge
Bacteroidia
Chitinophagales
Chitinophagaceae
Saprospiraceae
Sphingobacteriales
Cytophagales
Microscillaceae
Bacteroidales
Bacteroidetes_vadinHA17
Bacteroidetes_vadinHA17_ge
Flavobacteriales
Flavobacteriaceae
Gemmatimonadetes
Gemmatimonadetes
Gemmatimonadales
Gemmatimonadaceae
S0134_terrestrial_group
S0134_terrestrial_group_or
S0134_terrestrial_group_fa
S0134_terrestrial_group_ge
Firmicutes
Bacilli
Bacillales
Bacillaceae
Lactobacillales
Clostridia
Clostridiales
Armatimonadetes
Fimbriimonadia
Fimbriimonadales
Verrucomicrobia
Verrucomicrobiae
Opitutales
Pedosphaerales
Pedosphaeraceae
Pedosphaeraceae_ge
Acidobacteria
Subgroup_6
Subgroup_6_or
Subgroup_6_fa
Subgroup_6_ge
Subgroup_18
Subgroup_18_or
Subgroup_18_fa
Subgroup_18_ge
Acidobacteriia
Acidobacteriales
Solibacterales
Solibacteraceae_(Subgroup_3)
Bryobacter
Chlamydiae
Chlamydiae
Chlamydiales
Chlamydiaceae
Cyanobacteria
Melainabacteria
Obscuribacterales
Obscuribacterales_fa
Obscuribacterales_ge
Oxyphotobacteria
Chloroflexi
Ktedonobacteria
Ktedonobacterales
Ktedonobacteraceae
Chloroflexia
Chloroflexales
Anaerolineae
Anaerolineales
Anaerolineaceae
Anaerolinea
Caldilineales
Caldilineaceae
Dehalococcoidia
SBR1031
SBR1031_fa
SBR1031_ge
Actinobacteria
Actinobacteria
Micromonosporales
Micromonosporaceae
Frankiales
Micrococcales
Corynebacteriales
Acidimicrobiia
Thermoleophilia
Gaiellales
Spirochaetes
Spirochaetia
Spirochaetales
Spirochaetaceae
Spirochaeta_2
Omnitrophicaeota
Omnitrophicaeota_cl
Omnitrophicaeota_or
Omnitrophicaeota_fa
Omnitrophicaeota_ge
Patescibacteria
Saccharimonadia
Saccharimonadales
Saccharimonadales_fa
Saccharimonadales_ge
Parcubacteria

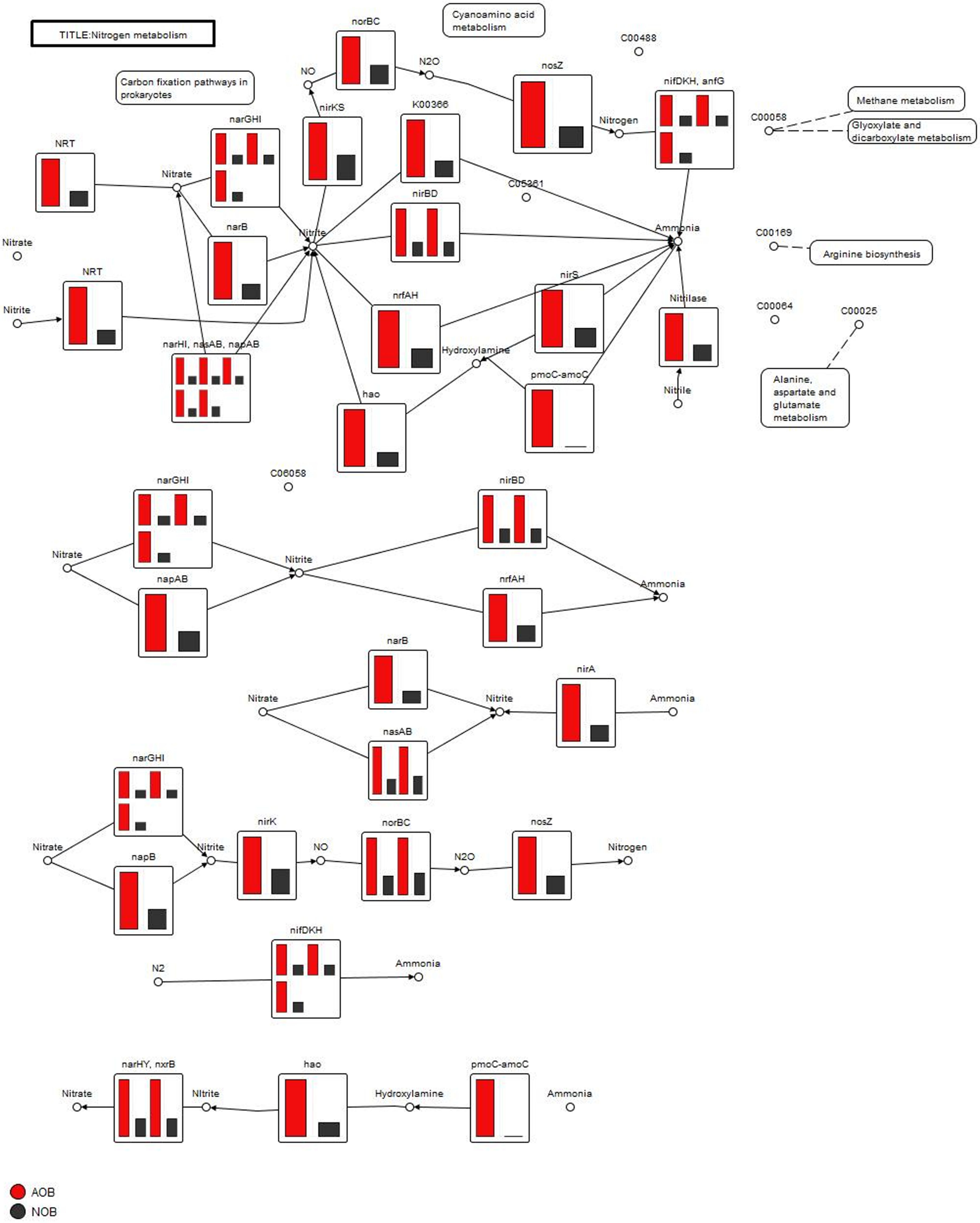
TITLE:Nitrogen metabolism
Cyanoamino acid metabolism
Carbon fixation pathways in prokaryotes
Methane metabolism
Glyoxylate and dicarboxylate metabolism
Arginine biosynthesis
Alanine, aspartate and glutamate metabolism
norBC
NO
N2O
nosZ
Nitrogen
nifDKH, anfG
C00488
C00058
nirKS
K00366
NRT
narGHI
Nitrate
C05361
nirBD
Nitrite
narB
Ammonia
C00169
nirS
nrfAH
Nitrilase
C00064
C00025
narHI, nasAB, napAB
Hydroxylamine
pmoC-amoC
hao
Nitrile
C06058
napAB
nasAB
nirA
napB
nirK
N2
nifDKH
narHY, nxrB
AOB
NOB

Microbial community profiling of ammonia and nitrite oxidizing bacterial enrichments from brackishwater ecosystems for mitigating nitrogen species

Viswanathan Baskaran[1], Prasanna Kumar Patil[1*], M. Leo Antony[1], Satheesha Avunje[1], Vinay T. Nagaraju[1], Sudeep D. Ghate[2], Suganya Nathamuni[1], Dineshkumar N[1], Shankar V. Alavandi[1], Vijayan K. Kizhakedath[1]

[1]Aquatic Animal Health and Environment division, ICAR-Central Institute of Brackishwater Aquaculture, Santhome
[2]Yenepoya Research Centre, Yenepoya University, Mangalore-575018

Corresponding Author: P. K. Patil,

Phone: +919941468356; Email: pkpatilvet@gmail.com; pkpatil@ciba.res.in

Table. S1. Taxonomy assignments of predicted genes encoding keyenzymes involved in nitrogen metabolism, detected in AOB and NOB enrichments.

Pathway	Genes	Phylum/Class	AOB consortium	Phylum/Class	NOB consortium
Dissimilatory Nitrate reductase	*NapA*	*Alphaproteobacteria*	*Rhodobacter, Bradyrhizobium*	*Plactomycetes*	*Unclassified Planctomyces,*
		Gammaproteobacteria	*Stenotrophomonas, Shewanella, Pseudomonas*	*Bacteroidetes*	*Arenibacter*
		Deltaproteobacteria	*Desulfobacca*	*Gammaproteobacteria*	*Unclassified Gammproteobacteria*
		Epsilonproteobacteria	*Sulfuricurvum*		
	nirB	*Actinobacteria*	*Janibacter, Verrucosispora, Kaistibacter, Arthrobacter*	*Actinobacteria*	*Unclassified Acidimicrobiaceae*
		Alphaproteobacteria	*Unclassified sphingomonadaeceae,Unclassified caulobacteraceae, Novosphingobium,Phenylobacterium, Unclaasifeid methylobacteriacee, Mathylobacterium sp, kaistobacter, Paracoccus*	*Alphaproteobacteria*	*Mesorhizobium, Novosphingobium, Anaerospora, Hyphomonas, Devosia*
		Betaproteobacteria	*Unlassified Methylophilaceae, Unclassified Comamonadaceae, Comomonas sp, Hydrogenophaga sp, Ramlibacter, Limnohabitans, Thiobacillus*	*Gammaproteobacteria*	*Unclassified Alteromonadaceae HB2-32, Alcanivorax, Unclassified Sinobacteraceae, Galcieola, Oleibacter*
		Gammaproteobacteria	*Psychrobacter sp, Pseudomonas, Methylomicrobium, Methylosarcina, Enhydrobacter, Alcanivorax, Acinetobacter, Marinobacter bryozoorum, Thiomicrospira*	Cytophagia	Unclassfified Cytophagaceae
		Bacteriodetes	*Sediminibacter*	*Candidtausphyla radiation*	*OD1-ZB2*
	nrfA/ C	*Bacteroidetes*	*Unclassified Caldilinea*	*Delatproteobacteria*	*Nitrospina*
		Epsilonproteobacteria	*Sulfuricurvum*	*Candidtausphyla radiation*	*OD1-ZB2*
				Planctomycetes	*Planctomyces sp.*
	NirD	*Acidobacteria*	*Ellin 6075, Candidatus solibacter, Candidatus koribacter*	*Acidobacteria*	*Candidatus solibacter, Candidatus koribacter*
		Actinobacteria	*Verrucosispora, Arthrobacter, Kaistibacter, Rhodococcus*	*Actinobacteria*	*Arthrobacter*
		Bacteroidetes	*Flavisolibacter, Sediminibacterium,*	*Bacteroidetes*	*Flavisolibacter, Sediminibacterium,*
		Firmicutes	*Alicycliphilus, Ammoniphilus.*	*Alphaproteobacteria*	*Sphingobium, Mesorhizobium, Hyphomonas*

		Alphaproteobacteria	*Caulobacter, Phenylobacterium, Sphingomonas, Nitrobacter*	*Betaproteobacteria*	*Hydrogenophaga*
		Betaproteobacteria	*Comomonas, Cupriavidus, Limnohabitans, Thiobacillus*	*Gammaproteobacteria*	*Alcanivorax dieselolei, HB2-32-31, Glaceiola, Halomonas, Marinobacter hydrocarbanoclasticus, Pseudomonas.*
		Deltaproteobacteria	*Anaeromyxobacter*	*Planctomycetes*	*Planctomyces, Gemmata.*
		Gammaproteobacteria	*Alcanivorax, Psychrobacter, Enhydrobacter, Marinobacter bryozoorum, Thiomicrospira, Paracocccus, Halomonas, Pseudomonas, Zobella.*		
		Verrucomicrobia	*Candidatus Xiphenimibacter*		
Assimilatory Nitate reductase	*nasB*	*Actinobacteria*	*Nocardioides*	*Actinobacteria*	*Nocardioides*
		Betaproteobacteria	*Thiobacillus*	*Betaproteobacteria*	*Thiobacillus*
		Gammaproteobacteria	*Bradyrhizobium*		
	nasA	*Gammaproteobacteria*	*Psychrobacter, Mainobacter*	*Planctomycetes*	*Planctomyces, Gemmata*
		Alphaproteobacteria	*Novosphingobium, Methylobacterium, Sphinogomonas, Kaistobacter, Paracoccus, Methylosinus.*	*Gammaproteobacteria*	*Unclassified Gammaproteobacteria, Alcanivorax*
		Betaproteobacteria	*Limnohabitans, Thiobacillus*	*Alphaproteobacteria*	*Mesorhizopbium*
		Epsilonproteobacteria	*Sulfuricurvum*	*Verrucomicrobia*	*Opitutus.*
		Firmicutes	*Exiguobacterium*		
	NirA	*Aplhaproetobacteria*	*Rhodoplanes*	*Aplhaproetobacteria*	*Rhodoplanes*
		Euryarchaeota	*Natranococcus, Natrialba*	*Planctomycetes*	*Planctomyces*
		Verrucomicrobia	*Candidatus Xiphenimibacter*	*Verrucomicrobia*	*Opitutus*
		Acidobacteria	*Candidatus Koribacter, Candidatus Solibacter*		
		Planctomycetes	*Planctomyces*		
Nitrification/Nitrogen transporter	*amoA/ pmoA*	*Betaproteobacteria*	*Candidatus* Nitrosomonas, Nitrosomonas marina		
		Archaea	*Nitrosopumilus,* , Nitrosphaerae, Nitrosococcus, Natranococcus,	*Betaproteobacteria*	*Nitrosospira, HB118. CPR OD1-ZB2*
	HAO/ HCP	*Euryarchaeota*	*Natranococcus*	*Planctomycetes*	*Unclassified planctomyces*
		Gammaproteobacteria	*Marinobacter, Paracoccus*		
		Betaproteobacteria	*Thiobacillus*		

	NRT	*Betaproteobacteria*	*Thiobacillus*	*Alphaproteobacteria*	*Thalassospira, Sphingomonas, Paracoccus, Hyphomicrobium*
		Gammaproteobacteria	*Stenotrophomonas, Psychrobacter, Marinobacter*	*Betaproteobacteria*	unclassified *Betaproteobacteria, Nitrospina*
		Alphaproteobacteria	*Sphingomonas, Paracoccus, Novosphingobium, Nitrobacter,Kaistobacter*	*Gammaproteobacteria*	*Alcanivorax, Unclassified Alteromondacea HB2-32-21*
		Epsilonproteobacteria	*Sulfurimonas, Sulfuricurvum*	*Nitrospirae*	*Nitrospira*
		Bacteroidetes	*Sediminibacterium*	*Verrucomicrobia*	*Opitutus.*
		Firmicutes	*Amminophilus sp.*	*Candidtausphyla radiation*	*OD1-ZB2*
		Euryarcheaota	*Natranococcus, Natrialba*		
		Planctomycets	*Planctomyces*		
		Actinobacteria	*Janibacter*		
	**AMT(Ammonium transporter)**	*Betaproteobacteria*	*Thiobacillus, Ramlibacter,*	*Alphaproteobacteria*	*Parvibaculum*
		Epsilonproteobacteria	*Sufuricurvum*	*Candidtausphyla radiation*	*OD1-ZB2*
		Alphaproteobacteria	*Rhodoplanes, Paracoccus, Nitratireductor, Nitrobacter, Nitrosomonas*	*Nitrospirae*	*Unclassified Nitrospira 4-29*
		Archaea	*Nitrosopumilus, Candidatus Nitrososphaerae, candidatus methanoregula, Candidatus Koribacter*		
		Firmicutes	*Desulfurispora*		
**Dentrification/Dissimilatory nitrate reduction/Nitrification**	*narG/ narz/nxrA*	*Euryarcahaeota*	*Natranococcus, Natrialba*	*Actinobacteria*	*Unclassified nocardioides*
		Alphaproteobacteria	*Paracoccus*	*Alphaproteobacteria*	*Anaerospora*
		Betaproteobacteria	*Limnohabitans*	*Gammaproteobacteria*	*Alcnivorax, Marniobacter.*
		Delataproteobacteria	*Desulfobacca*	*Nitrospirae*	*Nitrospira, FW4-29,GOUTA 10, HB118 , Unclassfied nitrospira*
		Gammaproteobacteria	*Marinobacter*		
		Epsilon proteobacteria	*Sulfuricurvum*		
		Nitrospirae	*Nitrospira, FW4-29,GOUTA 10, HB118*		

		Gammaproteobacteria	*Luteibacter, Marinobacter bryozoorum*	*Gammaproteobacteria*	*Unclassified Alteromonadaceae HB2-32-21, Alcanivorax, Glaceicola*
	narI	*Deltaproteobacteria*	*Desulfococcus*	*Alphaproteobacteria*	*Nitrobacter, Unclassified Hyphomonadaceae*
		Alphaproteobacteria	*Unclassified Rhodospirillaceae*		
		Betaproetobacteria	*Methylibium.*		
		Betaproteobacteria	*Unclassified betaproteobacteria, Hydrogenophaga, Thiobacillus, Methylibium*	*Chloroflexi*	*Unclassified caldilinea*
		Alphaproteobacteria	*Paracoccus, Pseudomonas, Hyphomicrobium, Unlassified rohodobacteracae, unclassified caulobacteraceae, Unclassified Physispharea*	*Gammaproteobacteria*	*Alcanivorax dieselolei, Glacieola, Halomonas, Unclasssifed Pseudomonas, Shewanlle*
		Euryarchaoeta	*Natranococcus, Natrialba*	*Nitrospirae*	*Nitrospira, FW4-29,GOUTA 10, HB118*
	narH/ narY/ nxrB	*Dealtaproteobacteria*	*Desulfobacca*	*Alphaproteobacteria*	*Nitrobacter, Paracoccus,*
		Chloroflexi	*Unclassified caldilinea*	*Betaproteobacteria*	*Thiobacillus*
		Crenarcahaeota	*Candidatus Nitrososphaera gargensis, Unclassified nitrososphaera*		
		Nitrospira	*Nitrospira, FW4-29,GOUTA 10, HB118*		
		Epsilonproteobacteria	*Sulfuricurvum*		
		Gammaproteobacteria	*Marinobacter sp.*		
		Alphaproteobacteria	*Paracoccus*	*Alphaproteobacteria*	*Parvibaculum, Nitratireductor*
		Betaproteobacteria	*Nitrosomonas gargensis, Unclassified Nitrosomonas*	*Bacterioidetes*	*Aequorivita, Arenibacter*
	nirK	*Gammaproteobacteria*	*Nitrosococcus, Thiobacillus, Luteibacter*	*Nitrospirae*	*Nitrospira*
		Nitrospirae	*Nitrospira, FW4-29,GOUTA 10, HB118*	*Verrucomicrobia*	*Opitutus*
		Euryarchaeota	*Nitrialba*		
		Verrucomicrobia	*Candidatus xipheminibacter*		
		Alphaproteobacteria	*Paracoccus*	*Alphaproteobacteria*	*Unclassified Alphaproteobacteria, Nitratireductor*
	norC/ E	*Betaproteobacteria*	*Unclasssifeid betaproteobacteria, Thiobacillus*	*Bacteroidetes*	*Arenibacter sp.*

		Gammaproteobacteria	***Unclassified Gammaproteobacteria, Nitrosococcus, marinobacter bryozoorum, ,***	***Betaproteobacteria***	***Arenibacter,***
		Epsilonproteobacteria	***Sulfuricurvum***	***Gammaproteobacteria***	**Paracoccus, Marinobacter sp.**
	nosZ	***Euryarchaeota***	***Natranococcus***	***Bacteroidetes***	***Aequorivita, Arenibacter, Unclassfied Flavobacterium.***
		Gammaproteobacteria	***Paracoccus, Marinobacter***	***Verrucomicrobia***	***Opituts***
		Betaproteobacteria	***Azovibrio, Thiobacillus.***	***Gammaproteobacteria***	***Marinobacter sp.***
		Chloroflexi	***Unclassified Caldiliniea***	***Alphaproteobacteria***	***Paracoccus***
		Chlorobi	***Unclassified Ignavibacterium***		
		Deltaproteobacteria	***Desulfobacca***		
		Bacteroidetes	***Sediminibacterium***		
		Alphaproteobacteria	***Paracoccus***		
